# The Adolescent Insomnia Questionnaire: the Turkish validity and reliability study

**DOI:** 10.1590/1806-9282.20240247

**Published:** 2024-08-16

**Authors:** Pınar Tekcan, Emine Erdem

**Affiliations:** 1 Erciyes University, Institute of Health Sciences, Child Health and Diseases Nursing – Kayseri, Turkey.; 2 Erciyes University, Faculty of Health Sciences, Department of Nursing – Kayseri, Turkey.

**Keywords:** AIQ, Reliability, Validity

## Abstract

**OBJECTIVE:**

This study aimed to evaluate the Turkish validity and reliability of the Adolescent Insomnia Questionnaire.

**METHODS:**

The study was carried out with 265 adolescents. Data were collected with the Adolescent Insomnia Questionnaire and the Cleveland Adolescent Sleepiness Questionnaire. Exploratory factor analysis and confirmatory factor analysis were used to analyze the construct validity of Adolescent Insomnia Questionnaire. The scale reliability was tested using test-retest, Cronbach's α test, Pearson correlation analysis, and inter-item correlation analysis.

**RESULTS:**

The Cronbach's α coefficients were found to be above 0.80 for all sub-dimensions and the total scale. Correlations between Adolescent Insomnia Questionnaire and Cleveland Adolescent Sleepiness Questionnaire scores were positively highly significant. The test-retest correlation analysis of Adolescent Insomnia Questionnaire was 0.675. The results of confirmatory factor analysis were χ^2^/df=2.861, comparative fit index=0.966, incremental fit index=0.966, Tucker-Lewis index=0.956, normed fit index=0.949, root-mean-square error of approximation=0.084. The suitability of the data for exploratory factor analysis was evaluated with Bartlett's test of sphericity (p<0.05), and the sample adequacy was evaluated with the Kaiser-Meyer-Olkin test (0.77).

**CONCLUSION:**

The Adolescent Insomnia Questionnaire Turkish version is a valid and reliable tool for measuring insomnia in adolescents aged 11–18 years. Adolescent Insomnia Questionnaire is a brief, practical, self-reported, age-appropriate, easily applicable, valid, and reliable tool in Turkish. This is the first Turkish validity and reliability study of Adolescent Insomnia Questionnaire.

## INTRODUCTION

Sleep, which covers one-third life of a human and is central to maintaining the health status, has an important place in the health maintenance of adolescents. Sleep problems experienced by adolescents cause life-threatening accidents and significant disorders in psycho-social functions^1^. In adolescents, insomnia is the most common problem among sleep disorders^2–4^. Notably, 7–40% of the general adolescent population appears to experience clinically classified insomnia. This rate varies according to characteristics such as population, age group, and gender^3^.

There are various tools for the assessment of sleep problems in adolescents^5^. These tools do not directly measure insomnia in adolescents. There is a critical need for insomnia tools that are validated for use with adolescents. However, there is no insomnia questionnaire for adolescents other than the Adolescent Insomnia Questionnaire (AIQ)^6^.

The AIQ was developed to measure the level of insomnia, and it consists of three sub-dimensions in adolescents^6^. Testing the AIQ by applying it to adolescents in a different country, language, and culture will give important results in terms of evaluating the screening sensitivity of the questionnaire. This is the first Turkish validity and reliability study of AIQ.

## METHODS

### Study design, sample size, and characteristics

This cross-sectional study was conducted between March and April 2022 in randomly selected three schools in the center of Amasya city in the Black Sea region. Data were collected from adolescents aged 11–18 years. The study was approved by the Amasya University Social Sciences Ethics Committee (04 January 2022, numbered: 50769). The research was conducted in accordance with the Declaration of Helsinki. Informed consent forms were signed by parents and children in the study. The sample size was required to be 10–20 times the number of scale items^7^, so the data were collected from 265 adolescents. A retest application of the AIQ Turkish version was conducted on 65 adolescents 15 days later from the first test. Data were evaluated with the IBM SPSS 20 statistical package program, and the confirmatory factor analysis (CFA) was determined by AMOS.

### Data collection tools

Demographic variables were collected from adolescents with demographic information forms. The AIQ is a 5-point Likert-type scale (0=Never to 4=Almost Always), which consists of 13 items and 3 sub-dimensions (factors 1, 2, and 3, respectively). Factor 1 is sleep dissatisfaction and impairments and has items 3, 5, 10, 11, 12, and 13. Factor 2 is sleep onset and has items 1, 4, 7, and 9. Factor 3 is sleep maintenance and has items 2, 6, and 8. Items 3, 4, 8, and 9 are reverse-scored in the AIQ. AIQ scores range from 0 to 52, and the higher the score, the higher the insomnia. The cutoff point of the AIQ was accepted as 15 points. To analyze the validity of the AIQ, the Cleveland Adolescent Sleepiness Questionnaire (CASQ) was used^5^.

### Validity and reliability stages of the questionnaire

#### Language validity

The AIQ translation was completed as forward and backward translations^8^. The original English-to-Turkish version was made by three researchers who were native speakers of both Turkish and English and had not seen the AIQ before. The Turkish-to-English version was translated by three researchers who were natives to both Turkish and English and had not seen the questionnaire before. The translation was completed by evaluating the suitability for Turkish.

#### Content validity

The content validity study was evaluated with the Davis technique in this study^9^. Experts from seven different fields reported that the AIQ Turkish version is suitable. The content validity ratio (CVR) and content validity index (CVI) were calculated by taking the opinions of these experts. CVR and CVI are determined as suitable (CVR: 0.87–0.99 for each item and CVI:0.95).

#### Construct validity

In this study, factor analysis is one of the commonly used methods to construct validity^10^. Kaiser-Meyer-Olkin (KMO) and Bartlett's test of sphericity (BTS), exploratory factor analysis (EFA)^11^, and CFA were carried out sequentially.

#### Reliability

Inter-item internal consistency was evaluated with Cronbach's α coefficient, Pearson correlation (PC) analysis, and inter-item correlation analysis.

## RESULTS

Of the adolescents participating in the study, 52.1% were male, 20.0% were in the ninth grade, and 76.9% had a nuclear family structure, and it was determined that the income of 49.8% of them was equal to their expenses. The mean age of the adolescents was 14.49±2.28 years. The mean scores of the AIQ and CASQ were 22.02±4.23 and 38.34±8.31 in adolescents, respectively (Table 1).

**Table 1:** Descriptive characteristics of adolescents.

Descriptive characteristics		n	%
Gender			
	Female	127	47.9
	Male	138	52.1
Class			
	5th grade	23	8.7
	6th grade	40	15.1
	7th grade	20	7.5
	8th grade	17	6.4
	9th grade	53	20.0
	10th grade	31	11.7
	11th grade	42	15.8
	12th grade	39	14.7
Family type			
	Nuclear	196	76.9
	Extended	43	16.9
	Fragmented	16	6.3
Income level (monthly)			
	Income less than expenses	35	13.8
	Income equals expenses	126	49.8
	Income more than expenses	92	36.4
		Mean ± SD	Med (min–max)
Age (years)		14.49±2.28	11–18
AIQ score		22.02±4.23	11–33
CASQ score		38.34±8.31	22–62

SD: standard deviation; AIQ: Adolescent Insomnia Questionnaire; CASQ: Cleveland Adolescent Sleepiness Questionnaire.

The internal consistency of all items of the AIQ Turkish version was good (Cronbach's α=0.82). Factor 1 was good in terms of internal consistency (Cronbach's α=0.83). Factors 2 and 3 were shown as excellent consistency (Cronbach's α=0.90 and Cronbach's α=0.93, respectively). The intraclass correlation coefficient (ICC) for the total AIQ score was found to be 0.822 and p<0.001 (Table 2).

**Table 2:** The validity and reliability results of Adolescent Insomnia Questionnaire.

Internal consistency analysis of AIQ									
Internal consistency						Cronbach's α	ICC	p	
AIQ total score						0.82	0.822	<0.000	
Sleep dissatisfaction and impairment subscale						0.83			
Sleep onset subscale						0.9			
Sleep maintenance subscale						0.93			
AIQ and CASQ correlation									
	AIQ			CASQ		Pearson correlation coefficient			p
	Mean	SD		Mean	SD	R			
	22.026	4.238		38.343	8.319	0.634			<0.001
Test-retest reliability analysis of AIQ									
AIQ		Test		Retest		Paired t-test results			
		Mean	SD	Mean	SD	Difference Test-retest	Difference	t	p
		22.098	3.948	23.114	3.768	-1.016	3.117	-2.547	<0.01

ICC: intraclass correlation coefficient; SD: standard deviation.

It was analyzed between the AIQ and CASQ total scores using PC coefficient analysis, and the criterion-related validity (CRV) was examined by calculating the correlation coefficients. It was determined that there was a high level of positive (r=0.634, p<0.001) significant correlation between the AIQ and the CASQ total scores. In the correlation analysis, there was a significant correlation between AIQ factors 1 and 3 and CASQ total score (r=0.580 and r=0.200, p<0.010, respectively) (Table 2).

The mean score of AIQ is not statistically different between the test and retest results. According to the PC analysis results, a high level of positive correlation was found between the total AIQ test and retest scores (r=0.675, p<0.001) (Table 2). These results indicate the reliability of the AIQ Turkish version.

According to the EFA analysis, it was determined that there was a three-factor structure in accordance with the original questionnaire model. The explanation rate of the model was determined as 71% in the EFA. The factor loadings were found like the original AIQ sample. The rotated factor loadings ranged from 0.53 to 0.96 (Table 3). The results of KMO (0.77) and BTS (p<0.05) indicate that the sample was sufficient for the EFA.

**Table 3:** Factor loadings in the three-factor model of Adolescent Insomnia Questionnaire (13 items).

Questionnaire factors		Rotated factor loadings
Item 3*	Factor 1 Sleep dissatisfaction and impairments	0.858
Item 5		0.836
Item 10		0.534
Item 11		0.676
Item 12		0.831
Item 13		0.650
Item 1	Factor 2 Sleep onset	0.575
Item 4*		0.964
Item 7		0.966
Item 9*		0.961
Item 2	Factor 3 Sleep maintenance	0.895
Item 6		0.947
Item 8*		0.956

* Item is reverse-scored.

To test the fit of the data to the model, CFA was conducted according to various fit indices such as chi-square/degree of freedom (χ^2^/df), root-mean-square error of approximation (RMSEA) with its 90% confidence interval (CI), comparative fit index (CFI), incremental fit index (IFI), normed fit index (NFI), and Tucker-Lewis index (TLI). It shows that the model is at an acceptable level when CFI, IFI, and TLI are greater than 0.90 (0.96, 0.95) and the RMSEA value of 0.08. The χ^2^/df ratio can be used as a measure of fit. It was determined that the χ^2^/df value is below the desired value of 3 (χ^2^/df=2.861; CFI=0.966; IFI=0.966; TLI=0.956; NFI=0.949; RMSEA=0.084). The CFA results of this study are accepted as excellent values. χ^2^/df, NFI, CFI, IFI, and TLI values show a perfect fit, while the RMSEA value shows an acceptable fit. The three sub-dimension structure of AIQ is confirmed according to CFA.

## DISCUSSION

In terms of both duration and quality, sleep is important for adolescent health. Unhealthy sleep during adolescence includes quantitative (short sleep duration, irregular sleep schedule) and qualitative aspects (night awakenings and difficulties falling asleep)^12^. These aspects lead to the development of insomnia. Although insomnia is the most common problem among sleep disorders in adolescents^3,4^, there is no scale/instrument other than the AIQ that directly measures insomnia in adolescents.

The short, easy-to-understand, and self-reported AIQ, which is suitable for the age and developmental level of children, can be used to determine insomnia. The AIQ cutoff score can be used to identify adolescents with insomnia during routine assessments of healthy adolescents. It is important to adapt the AIQ to different countries, languages, and cultures. The aim of this study was to assess the validity and reliability of AIQ on Turkish adolescents.

For content validity, CVR was calculated for each item of the questionnaire by taking the opinions of researchers who are experts in their fields. In line with expert opinions, the minimum CVR values vary according to the number of experts, but it is expected that the CVR value collected from experts will be higher than 0.50^13^. The CVR values of the AIQ Turkish version were determined to be higher than 0.875. Therefore, it can be said that the content validity of the AIQ was ensured.

It should be determined whether there are latent factors that should be done first in questionnaire development and adaptation studies^11^. EFA was used to determine the construct validity of the AIQ Turkish version. In this study for EFA, the concordance of the correlation coefficients between the variables was evaluated with BTS (p<0.05), and the sample adequacy was evaluated with the KMO test (KMO coefficient=0.77). The KMO must be higher than 0.50 for the adequacy of the sample size. Whether the questionnaire is suitable for factor analysis is evaluated by BTS significance (p<0.05). When the p-value is <0.05 for BTS, it is accepted that the questionnaire is relevant to the EFA^11^. It was determined that the AIQ Turkish version explained 71.76% of the total variance and a three-factor structure was obtained in the EFA. While the EFA was 69.21% and the factor loads were between 0.50 and 0.90 in the original questionnaire^6^, it was determined that the factor loads of the AIQ Turkish version items were between 0.53 and 0.96 in this study. These values are acceptable ranges^11^.

In this study, the three sub-dimension structure of AIQ was confirmed according to CFA. CFA was conducted according to various fit indices such as χ^2^/df, RMSEA with its 90%CI, CFI, IFI, NFI, and TLI^10,14–18^. When the model fit index, CFI, NFI, IFI, TLI, and RMSEA values of the AIQ Turkish version are examined, it is observed that the model is at an acceptable level^16–18^. The χ^2^/df ratio can be used as a measure of fit, and a ratio less than 5 is considered a good fit. The desired χ^2^/df value is below 3, and this value is found to be 2.86 in this study. The other fit indices scores were CFI=0.966, IFI=0.966, TLI=0.956, NFI=0.949, and RMSEA=0.084 in this study. χ^2^/df, NFI, CFI, IFI, and TLI values show a perfect fit, while the RMSEA value shows an acceptable fit. The CFA results of this study are accepted as excellent values according to the Measurement Models Fit Index and Accepted Values^19–21^. The fit indices in the original AIQ were RMSEA=0.097, CFI=0.92, and TLI=0.90^6^.

The relationship between the CASQ and the AIQ Turkish version score was examined using the Pearson product-moment correlation analysis, and the CRV was examined by calculating the correlation coefficients. It was determined that there was a high level of positive (r=0.634, p<0.001) significant correlation between the AIQ and the CASQ scores.

The Cronbach's α value for the total AIQ score was found to be 0.82 in this study. The Cronbach's α values of factors 1, 2, and 3 which are sub-dimensions of AIQ were found to be 0.90, 0.83, and 0.93, respectively. These results show that the AIQ Turkish version is highly reliable^21^. In the original AIQ study, while Cronbach's α of AIQ was found to be 0.91, the sub-dimensions of factors 1, 2, and 3 of AIQ Cronbach's α were found to be 0.87, 0.79, and 0.89, respectively^6^. The Cronbach's α of the AIQ Danish version was 0.88. The sub-dimensions of factors 1, 2, and 3 of Cronbach's α values in the AIQ Danish version were found to be 0.87, 0.84, and 0.73, respectively^22^. Besides, ICC was checked for internal consistency analysis of AIQ. While the ICC for the AIQ Turkish version total score was specified as 0.822 in this study, the ICC for the AIQ Danish version was 0.890^22^.

In the AIQ Turkish version retest, it was not found between the test and retest with the paired t-test analysis. A high level of positive correlation was found because of the PC analysis between the AIQ score and the AIQ retest score (r=0.675, p<0.001). According to the results of the AIQ which were performed by three different research groups in three different countries including this study, this questionnaire is an important tool for measuring insomnia in adolescents and can be used safely to test the clinical insomnia status^6,22^. However, further research is needed to determine all the features of the AIQ and to reveal its use in different countries and cultures.

## CONCLUSION

The findings of this study showed that AIQ is a valid and reliable tool for evaluating insomnia in Turkish adolescents aged 11–18 years. AIQ is a brief, practical, self-reported, age-appropriate, easily applicable, valid, and reliable tool in Turkish.
